# Book Reviews

**Published:** 1825-11

**Authors:** 


### CRITICAL ANALYSIS

OF

### ENGLISH AND FOREIGN LITERATURE,

RELATIVE TO THE VARIOUS BRANCHES OF

### Medical Science.

Quæ laudanda forent, et quæ culpanda, vicissim

Illa, prius, cretâ; mox hæc, carbone, *notamus.*—PERSIUS.

### DIVISION I.

### ENGLISH.

ART. I.—*Practical Remarks upon Indigestion; particularly as connected with Bilious and Nervous Affections of the Head, and other Parts: including Observations upon the Disorders and Diseases of the Stomach, and superior Parts of the Alimentary Canal. Illustrated by Cases.* By JOHN HOWSHIP, Assistant Surgeon to the St. George's Infirmary, &c.—8vo. pp. 192. London: Longman and Co. 1825.

THE work at the head of this article is not precisely what might be anticipated from the title: the first part, consisting of fifty closely-printed pages, being occupied with an account of certain affections of the throat and œsophagus. The arrangement adopted in describing these is not happy: we first have, in a succession of paragraphs, a sketch of the various diseases of the throat, &c. without any reference to their nature, (*e. g.* "relaxed uvula," "inflammation of the tonsils," "putrid sore throat," "chronic tumor of the tonsils," "inflammation from small-pox," &c. &c.); and then, in a continuation of the same set of divisions in paragraphs, we have the treatment of the diseases previously spoken of, following in similar succession. In this way, the history of relaxed uvula is begun in the first page,—then intervenes a great variety of subjects before we come to the treatment,—and, lastly, we have cases in illustration placed at the end; so that the account of the disease occupies three different portions of the volume, through which we have thus to hunt after it. Now, this arrangement, or rather this total want of arrangement, is bad, and ought to be reformed, as it detracts much from the interest which might otherwise be attached to the observations of the author,—gives to the whole an unconnected appearance, and renders the perusal of the work irksome and laborious. Instead, therefore, of following our author in the subdivisions above mentioned, we shall embrace under one head whatever useful information on any particular subject we may find scattered through the different parts of the volume.

The first disease which we find mentioned is a relaxed condition of the uvula, giving rise to various inconveniences, or even serious evils, from the irritation of the epiglottis and neighbouring parts; a particular account of which our readers will find in our review of M. LISFRANC's Essay on the Palate.* In addition to the tickling cough usually excited, our author informs us that in one case he has known a very tedious dyspeptic affection result from this apparently trifling cause. M. Lisfranc recommends excision in those cases which resist the usual application of astringent gargles. Mr. Howship, on the contrary, advises the removal of the part by ligature.

"The manner of operating," says Mr. H. "I have adopted, is to form a noose, large enough to receive the uvula, in the middle of the ligature. The patient's tongue being kept down, one end of the ligature, passed through the little ring at the extremity of a small steel instrument, held in the left hand, may be conveyed to its place, the noose being placed horizontally, so as to let the uvula drop through it; the other end of the ligature kept in the right hand, the noose may be tightened at the instant the uvula is perceived to be exactly included. A second knot being tied, and the ends cut off, the operation is finished. A very trifling degree of inflammation follows, the uvula shrinks, the ligature and part destroyed drop off together, and the part is completely healed within a week." (P. 27.)

The tendency to relaxation is not confined to the uvula, but occasionally extends to the surrounding parts: the tonsils, it is well known, frequently participate in this state of chronic debility, and even become much and permanently enlarged. Here, as in the preceding case, a decided preference is given to removal by ligature over excision. We are likewise informed that relaxation of the mucous membrane lining the parts, and connected with œdematous effusion into the cellular membrane behind it, "gives rise to polypous excrescences in the cavities of the nose, fauces, and œsophagus, as well as in the stomach and inferior parts of the intestinal canal; especially conducing, in the latter case, to the production of one of the most irksome and harassing infirmities to which the human body is liable—prolapsus of the rectum." We find nothing respecting the treatment of this œdematous condition of the fauces or stomach.

The affections of the lower bowels were spoken of by Mr. Howship in a former work.†

Among the various causes producing partial or complete ob-

---

* Vol. 50, page 512.

† *Practical Observations on the Diseases of the Lower Bowels and Anus.*

struction of the passage through the throat, may be mentioned tumors formed behind the mucous membrane of the fauces or gullet, and apparently originating in the cellular texture. A remarkable instance of this nature is given by our author.

"A specimen of this kind of disease, in the collection at St. Bartholomew's Hospital, exhibits a very large sarcomatous tumor, appearing to rise from between the coats at the posterior part of the pharynx. It projected forward towards the larynx, impeding respiration, and was at one time the subject of consultation for removal. The patient at length died, worn out by irritation. On examination, the tumor was found to have made its way upwards against the basis cranii, which removed by absorption, it continued to make its way, pushing the dura mater before it into the brain. To permit this intrusion, it was necessary that many ounces of the substance of the brain should be removed by absorption; and, notwithstanding this change took place, it is extremely curious that the mental powers remained perfectly undisturbed to the last." (P. 5.)

We pass over *putrid sore throat*, and the *inflammation from small-pox*, as containing nothing worthy of remark.

An observation of some interest is made in speaking of the suppuration which follows high inflammatory action about the fauces,—viz. that our author has seen several instances in which abscesses, formed either between the coats or close to the sides of the œsophagus, have broke into the canal, and ultimately left the functions of the parts unimpaired. The following case is related in illustration:

"*Abscess of the Œsophagus.*—February 10, 1822.—I was called up during the night to see a young woman, S. N., who six weeks before had awoke suddenly in the night, with acute pricking pain in the throat, in a spot exactly opposite the top of the sternum. The pricking pain was greatest if she attempted to swallow a little fluid: her sensations were those of a swelling, threatening suffocation. With so much difficulty in breathing, she passed a very bad and sleepless night. The next morning better; she was able to eat her breakfast pretty well.

She now remained well till, on the evening of February 10, she felt her throat rather sore. During the day she had swallowed with perfect ease, but awoke at midnight with a feeling of impending suffocation, from a sense of fulness confined entirely to that part of the throat before affected. In this point, excessively painful to the least external pressure, she felt a violent, acute, prickling heat. The easiest position was sitting up, at perfect rest: when she lay down, it felt to rise in the throat, with aggravated difficulty of breathing. The voice was impaired, and the exercise of it painful. No material affection of tongue or skin. Pulse rather hurried, with much anxiety. An anodyne draught was directed, with little effect.

February 10, nine A.M.—Symptoms unaltered; had passed the night sitting up. About noon to-day, she endeavoured to swallow a little tea. It did not pass lower than the swelling, where it seemed to move or roll about, and then was rejected, with violent sickness and straining; which brought off some thick yellow matter, evidently purulent, with other fluid, from the stomach. A sense of immediate and perfect relief, both from the swelling and pain, the moment she was sick, naturally suggested to her the idea that something had broken; for she could now lay down with comfort, and slept an hour. In the course of the afternoon I directed her some castor-oil, which operated thrice.

"February 11.—After resting well through the night, she felt herself much better.

"February 12.—Could swallow with ease and comfort, and considered herself in every respect restored to perfect health.

"November, 1824.—Upon inquiry, I was happy to hear there had been no return whatever of the above complaint." (P. 149.)

In the course of the present article, we have dealt in analysis rather than in criticism, and have extracted passages which have, perhaps, little of novelty to recommend them, although so much of verity as occasionally to border upon truisms. There is, however, one proposal which, whatever it may have of accuracy, appears at least to have the merit of being new. It is as follows:

"Where, from the accidental and temporary detention of any small substance in the throat, there is, from the circumstances noticed in a preceding paragraph, reason to apprehend the formation of a pouch in the pharynx or œsophagus, the first object must be to ascertain the precise seat and extent of the change. For this purpose an œsophagus-sound, with a ball of one-fourth of an inch in diameter, may be very lightly and gently passed into the throat. With a view to determine on which side of the canal the duplicature, or little pouch, exists, the passage must be examined in the same manner in which the urethra is directed to be sounded, for ascertaining the situation and direction of a false passage in stricture. The ball must first be made to glide lightly down in contact with the posterior surface of the canal: if this fails, the instrument is to be withdrawn, and passed down lightly in contact with the anterior side of the canal; and in the same manner the lateral surfaces are to be successively traversed, until the ball of the sound, being in the same line with the pouch, is caught by the obstruction. The true nature of the obstruction may be in another way demonstrated, by introducing a large-sized ball, which, if of the diameter of an inch, will, in the early stage of the complaint, pass down without perceiving any difficulty at all; while the smaller ball will be invariably stopped.

"It is sufficiently clear that, provided the patient has not in the first instance neglected his own case, the surgeon will, by the above examination, be enabled to ascertain that the recess or pouch is of small extent, as to breadth and depth; and it may, consequently, be fairly concluded that as yet the internal membrane only is concerned in its production. The situation of the pouch ascertained, its depth may be determined with sufficient accuracy, by first passing the instrument lightly to its bottom, then leaning the ball lightly over against the opposite side of the canal, and gently withdrawing it; observing the distance passed before the ball springs from its confinement; or, in other words, before it slips over the edge of the pouch. By this means, unless the affection has been unguardedly allowed to take its own course for many months, the depth of the recess will most probably be found to be within half an inch.

"Now, as the increase and eventually dreadful consequences of a pouch thus situated, are entirely owing to the lodgment of food within its cavity, it is evident that the only care required will be to render it, in the early stage of its formation, incapable of its office. If within reach, this might be effectually done *by snipping it through the middle, to the bottom, with a pair of scissors: and the same operation may, with equal security and certainty, be performed in the pharynx or upper part of the œsophagus, by a well-made instrument, properly constructed for its accomplishment.*

"The œsophagus-sound, of strong silver wire, adequately curved, and carrying a ball of a quarter of an inch diameter, may be adapted for this operation by the addition of a small probe-pointed blade, about an inch in length, connected to the sound by a hinge; its probe-point shutting into a notch in the ball, and its smooth back forming an even line, from the face of the ball backward to the wire of the sound. By the application of a small stiff spring to the blade, and the addition of a second moveable wire to the first, the wire may readily be made to raise the probe-point of the blade from the ball about half an inch, and the spring to close it again at pleasure. The instrument, however, should be so well constructed, that, while the spring has sufficient power in closing the blade to divide perfectly and promptly the interposed membranous fold, the movements of opening and closing the blade should take place with the greatest possible ease, and the least possible friction.

"Suppose, then, the pouch or sacculus to have been discovered upon the posterior side of the pharynx, the instrument just described must be so constructed that the blade shall stand upon the convex side of the curve of the sound. In performing the operation, the patient's head held steady in a good light, and the tongue depressed by an assistant, the sound is to be passed down lightly in contact with the back of the throat, till caught in the little pouch; the ball is then to be gently raised out of the pouch, and by the moveable wire the blade is to be opened, and the instrument in this position again passed downwards: the probe point will be immediately felt to have reached the bottom of the pouch, and the wire now left at liberty, the spring again closing the blade, the membrane is divided, and the operation finished.

"The complete success of the operation may be ascertained by passing a small and slightly curved elastic bougie, keeping it in gentle contact with the back of the canal. Before the operation, the point would thus have been caught in the pouch, but now the obstruction will not be perceived. This point determined, the bougie may be passed once a-day for a fortnight, to prevent the possibility of any re-union." (P. 40—42.)

We were rather startled at perusing this quotation, and have placed in italics that portion which appears to us most objectionable. Is it really true that an operation of this kind may be performed with "security and certainty?" We are always unwilling to condemn on our own poor judgment, and are of all others the most sceptical of editorial infallibility: we therefore proposed the question to several surgical friends, who expressed as much astonishment at the passage as we had ourselves experienced. One, indeed, remarked that the author did not say the operation could be done with security or certainty, but only that, of two operations, one might be done with *equal* security and certainty, &c. We know not whether Mr. Howship will thank him for this very ambiguous defence; but we would ask, has it ever been done? Is not the recommendation likely to do harm, by inducing young and inexperienced men, who may regard the work before us as one of authority, to attempt a hazardous and difficult operation. Every operation must be difficult which is performed in parts which we cannot see; and none can be free from hazard where a cutting instrument is introduced into a part so situated as the œsophagus.

The causes of *impeded deglutition*, enumerated by practical writers, are successively gone over by our author, but without any details worthy of being extracted. The distress occasioned by *flatus* in hysterical women, is too common to have escaped the notice of any of our readers. We have never ourselves had occasion to witness any thing so formidable as a case which occurred to Mr. Howship.

"Some time since I had an opportunity of witnessing a most unusual degree of this kind of difficulty and distress, in a thin, middle-aged woman. Somewhat exhausted by walking when I saw her, a severe paroxysm of aggravated distress came on. The extreme distention of stomach, raising the whole abdomen, almost prevented her drawing any air into the lungs. The rapidity with which the gas was formed, almost exceeds credibility. The air escaping from the stomach was generally expelled in a forcible loud rush, which continued for the space of half a minute, liberating, perhaps, a volume equal to several pints. She was then able to breathe again, till, in a minute or a minute and a half, the same imperious necessity for breaking off more wind returned as before. During the paroxysm, the quantity of air evolved, judging from the successive volumes set at liberty, must have been immense; as, for near an hour, the average that rushed up from the stomach was, I conceive, at least equal to a pint every two minutes. As long, however, as the œsophagus remained passive, the distress was comparatively small; but now and then the canal was closed by spasm, preventing the escape of the flatus collected below, and thus occasioning a struggle, severe beyond description, attended with all the convulsive appearances of actual suffocation, for the space of nearly half a minute; and, even when relieved by the spasm giving way, being instantly succeeded by a long and continued rush of air from the stomach, the relief to the breathing was scarcely perceptible." (P. 15.)

In cases of *spasmodic stricture*, the use of the bougie is recommended.

The most formidable cause of stricture, however, is schirrus of some part of the œsophagus; and the burning pain of the part itself, with the inability to swallow, the obstacle presented to a bougie, and the emaciation of the patient, are sufficient to mark the nature of the disease. We are informed that Mr. Heaviside regards the pain shooting towards the ears in strictures situated high in the œsophagus, and, in those situated lower down, towards the shoulders, as very unfavourable, and indicative of cancerous affection.

A singular and very distressing case of *obliteration of the œsophagus*, is mentioned in one of the French Journals,* and which, although not in the work before us, we may briefly describe. A young man was admitted into the Hôtel Dieu at Lyons, having lost the power of swallowing, in consequence of taking a quantity of nitric acid, which had brought on inflammation of the œsophagus. For some time, fluids were introduced into the stomach through a hollow gum-tube, until at length even a sound could not be passed, and he was nourished by enemata. The patient from this time suffered dreadfully from hunger, became rapidly emaciated, and died at the end of ten days from the period when the stricture became entirely impervious, and two years from the date of his swallowing the acid. On opening the body, the œsophagus was found obliterated for four inches, by adhesive inflammation gluing its sides together.

In terminating the diseases of the throat, the author mentions in a note an example of *imperforate œsophagus*, adding, that he does "not know of any similar observation." Two such have been recorded in the pages of this Journal during the present editorship: one occurred to a practitioner at Walworth, and the preparation was given to Sir A. Cooper;† and the other is mentioned by Dr. Sonderland, of Bremen.‡ It is remarkable that in both alvine evacuations took place. Of the case alluded to by Mr. Howship, it is merely mentioned that the stomach contained a glairy mucus, and the bowels the usual meconium.

Part II.—This portion of the work professes to treat of the disorders that more immediately regard the Stomach, and is preceded by a comparative view of the function of digestion, "as traced upon the ascending scale of animal life." As this, however, comprehends nothing but what is contained in every elementary treatise of a similar kind, we shall take leave to pass it over.

---

* *Bulletin de la Societé d'Emulation de Paris*, Juin 1823.

† *London Medical and Physical Journal*, vol. xlix. page 10.

‡ *Med.-Chirurg. Zeitung*, 1822.

Mr. Howship, it seems, has suffered much from *sea-sickness*, and is of opinion, with every other person who knows any thing about the matter, that it is "a sympathetic affection dependent on a disturbed state of the brain." Unquestionably, remaining perfectly quiet and motionless affords the greatest comfort we are capable of obtaining in this horrible affection, although various remedies have been recommended. From the quotation which follows, our readers will perceive that fright seems to be a most effectual cure.

"Various means of relief have obtained credit under this affection of stomach. Taking wine will often succeed: spirits and water I have frequently known answer the same purpose; and have also seen it completely checked for the time by eating two or three fresh apples. It appears also that any strong impression upon the mind, as exerting a powerful influence on the brain, is capable of at once arresting the progress of this complaint. On one occasion at sea, in heavy weather, I was myself extremely ill, sick, and lying down: a sudden noise and commotion upon deck drew my attention, when one of the officers, running into the cabin, told me the hold of the ship was all in flames. The more powerful impression in an instant took the lead: I jumped up; and although, almost by a miracle, the fire was extinguished, and the ship and all the lives providentially saved, I from that moment felt neither headache nor sickness." (P. 113.)

Injuries of the head from external violence, it is well known produce sympathetic derangement of the stomach, generally attended with sickness and vomiting; but, in those arising from chronic inflammation of the membranes of the brain, with purulent effusion, our author has usually seen a suspension of its functions, but without sickness.

Derangement of the stomach from acute disease within the chest, is less common than most other sympathetic disturbances of this organ; but all abdominal diseases exert a great influence over the stomach. This is too well known to require any elucidation.

As the disorders of other parts affect the stomach, so the diseases of this viscus powerfully affect almost all the other organs. The head is the part first spoken of in the work before us, and a well-known fact is pointed out—that giddiness, headache, and other symptoms of a similar kind, frequently proceed from derangement of the stomach.

"In one of these cases, a gentleman had, for this complaint, long been in the habit of frequently losing blood by cupping, under the direction of his physician, and felt at last as uneasy at the idea of the remedy as of the disease. By a course of medical and surgical treatment, to restore power to the stomach and action to the bowels, the complaint in the head was perfectly removed; and from that time to this (six years) he has had, I believe, no return. In another, which, in severity at least, was similar to the above, the correction of habitual derangement of the stomach and bowels, and the removal of a disease from the lower extremity of that canal, entirely cured a complaint in the head of many years' standing, which had frequently confined the patient to his bed for days together." (P. 61.)

Now, we nothing doubt the truth of all this, and are satisfied that Mr. Howship, who cured the patient, treated him better than the physician who did not: but, after all, the reader gains little practical information, unless he be made acquainted with the circumstances in the case which led our author to discover that the stomach was primarily in fault. Two persons shall complain of pain and giddiness in the head, with some derangement of stomach : one of these gets well from the use of purgatives, and the other does not, but recovers on being cupped. This is a matter of every-day occurrence; and if our author, in tracing the phenomena of digestive complaints, had pointed out by what means we could discover, *a priori*, the affections of the head which would yield without the loss of blood, he would have conferred an essential service on us, who every day find occasion to doubt the diagnostic marks contained in practical writings.

The sympathies between the thoracic viscera and the stomach are scarcely less frequent, and perhaps not at all less distressing; yet we cannot agree with our author when he says—

"Many individuals are habitually subject to violent palpitation at the heart, *and in every such instance, of which I have been able to trace the history or watch the progress, it has appeared to be the result of derangement in the digestive functions*, judging from the irregular and generally torpid state of the bowels which has attended, and particularly from the great relief, if not perfect removal, of the distressing pectoral symptoms, always obtained by directing the attention to the feelings and functions of the abdominal viscera." (P. 62.)

That every case of palpitation at the heart has been produced by derangement of the chylopoietic viscera, is a position too exclusive to be admitted. It is true that one of the most distressing symptoms attending dyspepsia, is uneasiness and oppression in the chest, with fits of palpitation ; and, on the other hand, in primary organic diseases of the heart, very frequently many of the most troublesome symptoms of indigestion are present, particularly flatulence ; and such cases, although not altogether dependent on the state of the stomach, are nevertheless much aggravated by its derangement: and it is in such instances of morbid action of the heart brought on by dyspepsia, or of dyspepsia sympathetic of organic disease of that part, that benefit has appeared to us to arise from the use of the prussic acid. At all events, we agree with Mr. Howship that what relieves the stomach most—most relieves the heart.

Various other sympathies are pointed out.

"In truth, I believe there is no derangement of function or structure, however remote, but may be influenced, and frequently within half an hour be changed in all its feelings, by slightly modifying the nature of the contents, thus qualifying the results of chemical and vital action, in the stomach. I am intimately acquainted with a gentleman, who, from a blow on the leg, has for some years had a swelling on the shin. His own feelings have taught him that if, while free from any local uneasiness, he eats the least bit of unripe orange, or even a ripe grape, he is sure, within ten minutes, to feel pain in the swelling upon the leg, which continues to be troublesome for the rest of the day." (P. 63.)

A little further on we find an account of the evils resulting from the abuse of *fermented liquors;* and this we shall quote at length, both because the subject is interesting, and because the author, in his Introduction, especially directs our attention to it.

"In those destructive complaints induced by intemperance in drinking, we see that the earliest ill consequence of continued excess, is a declension of appetite, which goes on to a total want, not only of desire for food, but of power to retain it. This exhaustion of power is so complete, that frequently nothing will stay on the stomach but ardent or spiced spirits. All healthy function superseded, even should food be occasionally retained, it is left to undergo the ordinary changes that warmth and moisture would produce out of the body. In passing the small intestines, a spontaneous process takes place, usually productive of flatulency, and often running on to the acetous fermentation; and, during its residence in the great intestines, still further blended with the depraved secretions of the canal, and still further advanced in decomposition, the whole mass conveys so much irritation to the already-excited and too-irritable cavity of the canal, as not unfrequently induces extensive ulceration in the bowels. These consequences I have in several instances traced after death.

"Where a long course of excess ends fatally, it often happens that the destruction of the digestive powers is followed by deranged functions or diseased structure in the liver. This disease of liver, as far as I have observed, has generally had the appearance of an interstitial deposit into the substance of the gland, somewhat resembling a scrofulous or cheesy matter, most commonly forming either very numerous small tubercles, of a light or bilious tinge, or sometimes exhibiting large tubercles or tumors, few in number. In the first case, there is usually a general contraction and increased firmness of the whole substance of the gland, appearing to depend on a morbid state of the connecting cellular tissue of the viscus; in the second case, where, from the excess of interstitial deposit, the mass of the liver is greatly increased, the diseased condition of the cellular tissue is very demonstrable, with conversion into fasciculated and ligamentous fibres, nearly resembling what happens in cancerous disease. A singularly perfect specimen of the latter variety of disease is preserved in the morbid anatomical collection of Dr. Hooper.

"In cases where, from intemperance, the loss of energy in the stomach has led to ill consequences more immediately depending on the habitually excessive excitement of the nervous powers, delirium of a peculiar character, and oppressed brain from a peculiar cause, progressively ensue. Under these circumstances, also, if the complaints are not cured, the event is fatal. But what do we learn by diligent examination after death?—that the ultimate consequence of long suspension of the digestive process, is an absolute deficiency in the necessary supplies of nutritious matter to the vascular system; and that, instead of the proper constituents of healthy blood, the vessels, large and small, especially those within the head, contain a fluid which, in the transparent veins of the brain, appears like water scarcely tinged, and in the arteries is pale and thin, from the great deficiency in the quality and quantity of crassamentum.

"It occasionally happens that affection of stomach from excessive drinking, is not confined to suspension of its healthy functions. I have attended in one case where, after being subject many years to frequently returning attacks of burning heat at stomach, irritability, and vomiting, the patient died rather suddenly, from chronic inflammation having terminated in a general effusion of purulent and coagulable matter into the cellular texture between the coats of the stomach. There is reason to believe that chronic excitement from this cause has, in many instances, laid the remote foundation of disease in the stomach, which has eventually become cancerous." (P. 67—70.)

"In tracing the consequences of dyspepsia from drinking, it was observed that the stomach not only loses the power of reducing the food by digestion, but its faculty of retention also. Should the power of retention be only partially destroyed, it becomes important to consider the principle on which, under that state of the alimentary canal, the most nutritious aliment, instead of being subservient to the purposes of support, may prove a source of misery to the unhappy patient; running into decomposition, and sometimes exciting in the bowels incurable disease.

"One of the principal difficulties in the treatment of these complaints is, that the propensity to continue the destructive habit of excess is frequently so strong, that the patient has not resolution to break through it. As regards opinion, it should, on another ground also, be sometimes doubtful; it being frequently impossible to estimate correctly the prospect of success, even under strict observance of rule and restriction, from the occasional uncertainty of symptoms.

"A most important question appears to be, whether it is proper or safe to lay aside at once that stimulus which has excited and kept up the disturbance. It appears to me that, if it can be done with safety, there can be little doubt of its propriety; although it may, on the other hand, be urged that, by entirely withholding that power by which the constitution was sustained, the patient must sink into excessive exhaustion.

The fact, however, is, I have seen several, and detailed two instances, in which, the disturbed state of the brain and stomach being considerable, I repeatedly laid unqualified interdict, prohibiting any thing fermented; and in each instance, on each occasion, the patient did perfectly well. I am aware that some physicians, for whose talents I have the highest respect, qualify this prohibition: but reference to the examples now given will suffice to prove the perfect safety of adopting a more decided rule; and, from what I have seen in the practice of others, I believe recovery is more rapid under a complete than under a partial abstinence from fermented liquors.

"Where the excitement has been extreme, the irritability of the stomach scarcely permits it to receive solid or fluid. Under these circumstances, the most advisable mode of treatment appears to be the exhibition of bark with opium. Provided the decoction and tincture of bark are so combined with the tincture of opium as to produce some little influence upon the stomach, the medicine being given in the quantity of a table, dessert, or even tea spoonful, it will scarcely excite vomiting: this, repeated every half-hour, will soon enable the stomach to retain some warm milk or beef-tea, given in the same restricted and cautious manner. In this way nourishment may be taken, with an advantage that will soon be sufficiently manifest. As the treatment proceeds, the tonic continued, the anodyne power must be gradually lessened, and, if needful, an aperient eventually substituted. By two or three weeks' perseverance in this plan, the patient's health will commonly be essentially improved, if not entirely restored.

"When habitual pain or tenderness in the region of the liver, uneasiness about the shoulder, or bilious tinge upon the skin, affords reason to expect disturbance in the hepatic functions, occasional small doses of mercury internally may, with advantage, form a part of the treatment: should acute local pain supervene, tonic medicines must immediately be laid aside, and perhaps give place to an opposite line of treatment, to be regulated from day to day by the state of the pulse, tongue, and other circumstances that may indicate a tendency to inflammatory action.

"Accidental facilities for observation have led me to consider very attentively the influence of this state of stomach and system, in exciting sympathetic disturbance in the functions of the brain. Where this disturbance is considerable, the character of the delirium is too peculiar to be mistaken; and when a patient can be depended on, or can be prevented from taking what would be improper, the treatment suggested will, I believe, very rarely fail to relieve and remove the complaints; unless where comatose drowsiness, or symptoms of paralysis, have already taken place, when the event of the case must, of course, be very uncertain.

"In answer to what I have ventured to advance regarding the influence, or even the existence, of an impoverished condition of the blood, as observed in examining the brain under this state of constitution, it may perhaps be objected that I attribute too much to the fluids, and too little to the solids. It is in some degree matter of opinion; but it is nevertheless founded on observation, and, I think, therefore on truth.

"As far as I have traced the consequences of this complaint, it appears that, so long as the injury to the general or local health falls short of the production of actual organic disease, the facility with which the constitution will recover itself, if only two conditions, correction of the habits and proper treatment, are afforded, is often astonishing. Sometimes it is true that in the stomach, as in other organs, the pernicious influence of excess runs beyond mere derangement of function; but, as regards that particular instance, the impression on my mind, from the symptoms and appearances, was that, had the long-established habits of intemperance been laid aside only a week previous to the fatal event, the stomach might probably have recovered itself, and, under more regular habits of life, would most likely have remained healthy." (P. 129—132.)

It is remarked that there are various affections of the stomach independent of inflammation, although at least equally painful. These are supposed to depend upon irritation, producing spasm; an opinion which derives plausibility from the manner of the attack, which consists of paroxysms, and from the fact of its frequently yielding to antispasmodic remedies. The most severe and formidable examples of this kind are those met with among gouty patients, although the actual existence of a spasmodic contraction of the muscular fibres, both in this and other cases, may be questioned: at least, we are not aware of any demonstrative evidence upon the subject. The author before us, who throughout refers with much deference to the opinions of Dr. Hooper, favours us with his views on this occasion. He does not conceive that any spasmodic affections of stomach, referable to gout, can ever be traced after death as such; the stomach, under such circumstances, being usually more or less filled with flatus and relaxed; and, although a person free from retrocedent gout may be seized with violent and terrible pain, which he refers to the stomach, and which has suddenly proved fatal, yet has this viscus been uniformly without contraction, exhibiting only patches of increased capillary vascularity. On mentioning the subject to Dr. Baillie, he expressed a similar opinion.

The usual treatment is recommended, consisting of ammonia, opium, ether, &c.

From the stomach, our author next passes on to the diseases of the Liver and Spleen; but, as he again returns to the stomach some fifteen or sixteen pages further on, and as we prefer finishing one branch of the subject first, we shall take leave to reverse the order of this arrangement.

*Polypus of the stomach* is alluded to as of very rare occurrence; and it is sufficient for us to mention it, as there are no symptoms by which its existence can be at all determined during life: "complaints of frequent pain and sickness at stomach, with] an obstinate and irritable state of bowels, with diarrhœa," which are mentioned by the author as leading him to think that the "probability of it may be suspected," being common to too many affections of the chylopoietic viscera to be of any assistance.

*Ulceration of the stomach* is next brought under our notice, and the following cases are worthy of perusal:

"In 1803, I noted an examination, made with Dr. James, of the body of a male infant, five months old; only for a week subject to fits of pain, with mucous and slimy laxity of bowels. No sickness at stomach, but said to have ate bread and milk greedily to the last. On opening the body, the intestines were healthy: on the surface of the stomach were numerous small dark spots, which, on opening the cavity, were found to depend on so many small ulcers, no larger than grains of millet-seed; these, destroying the villous and muscular coats, had left the peritoneal covering thin and transparent. There was not the least trace of redness or induration around the ulcers, the stomach appearing otherwise healthy.

"The other instance occurred in a grown person. In 1814, I examined, with Dr. Merriman, the body of E. N., a young woman who died in advanced pregnancy, without any known cause. The only remarkable appearance was in the stomach, in which was much flatus and some fluid contents. On its external surface, near a dozen small dark-coloured spots appeared. These spots, raised above the surface, subsided readily when touched, as if the coats of the stomach were weakened. On opening the cavity, the villous membrane, like the peritoneal covering, was in a natural state, except in the small spots: the largest, near the lesser curvature of the stomach, rather less than a split-pea in diameter. These small ulcerated spots, destroying the villous and muscular coats, derived their colour partly from the transparency of the membrane, and partly from a sloughy tendency induced in the cellular tissue of the peritoneal covering. There was not the least trace of inflammation or thickening in the coats of the stomach, the ulcers looking as if cut or scooped out of the healthy surface." (P. 97, 98.)

In the former of these cases, the ulceration had destroyed the two internal coats, leaving only the peritoneum; and, in the latter, it appeared "as if the coats of the stomach were weakened."

Much interesting information on the softening and ulceration, or even rupture, of the stomach, will be found in a paper by Dr. Gairdner, in the Transactions of the Medico-Chirurgical Society of Edinburgh; in a work by Cruveilhier,* reviewed in the 48th volume of this Journal; and in a paper by Mr. North, in the 52d volume. This very peculiar form of disorganisation would have formed an interesting subject for discussion in the work before us, but it seems to have escaped the notice of the author.

---

* *Médecine Pratique, &c.*

Various cases of *cancer of the stomach* are detailed, principally from the works of other writers; and particular mention is made of the hemorrhagies sometimes accompanying them. This part of the volume terminates with an account of some cases in which individuals had swallowed boiling water. Our readers may remember that Dr. MARSHALL HALL, in his paper in the Medico-Chirurgical Transactions (vol. xii.), has regarded the symptoms as indicative of inflammation of the glottis and larynx,—not the stomach; the progress of the fluid being, according to his idea, arrested by a spasmodic action of the throat. In this Mr. Howship does not agree with him, and relates two cases, which he thinks invalidates Dr. Hall's opinion. Let our readers judge.

"Mary Stockman, aged two years, April 12, 1822, at three P.M. went to the tea-kettle, and drank out of it some water boiling hot, which had been removed from the fire not five minutes before. She screamed out, and ran to her mother: some she threw out of her mouth, but some, it was clear, had been swallowed, as she immediately put her hand on her stomach, which was afterwards the principal seat of pain and distress. The child was immediately brought to me, with flushed face; rapid, failing, and irregular pulse; breathing laboriously; and with every appearance of approaching death. A gentle aperient medicine, with some liquor acet. ammon., was directed at short intervals, with immersion in the warm bath. All the afternoon, writhing with agony, no one could nurse her; but she took the medicine, kept it down, and had a motion towards the night, which passed without sleep.

"April 13.—Pulse weak and intermitting, about 120. An oily aperient mixture was now directed; and operated twice during the following night.

"April 14.—Respiration still hurried and laborious; pulse much the same. Much less expression of pain on gently pressing the præcordia. A saline draught, a small blister to the stomach, and castor-oil at night, were directed.

"April 15.—I found the skin cool; less apparent anxiety; breathing much quieter, and more free; pulse more regular. Had more sleep the preceding night, and less general restlessness. The bowels had been moved twice. The medicines were continued.

"April 16.—Pulse quite regular, 120; restlessness nearly gone. Had suckled for the first time since the accident. The bowels were regulated by castor-oil. The child was so much better, that I only visited her April 21, and then found her restored to perfect health."

"On the afternoon of August 30, 1824, calling in to visit another patient, I was requested to see Henry Mills, a boy four years old, said to be dying. At six P.M. the preceding day, playing near the fire, the child (in the habit of drinking in that way,) had drawn some boiling water out of the spout of the tea-kettle. The nurse, alarmed, ran to the nearest apothecary, who gave the child an emetic, assuring the person there was not the least danger. Violent retching followed; but nothing else was done, till next morning Mr. Hamerton was requested to visit the child, who very properly directed a warm bath, some leeches to the throat, and an aperient mixture; acquainting the parents that, so far from there being no danger, there was scarcely any chance of recovery. When I saw him, he breathed very quick and anxiously, with a skin cold and clammy, and a pulse hurried and intermitting. In a moment he started, sat up, and looked wildly about; and at eight P.M. died.

"The following day leave was obtained to examine the body. The stomach externally had no appearance of inflammation: on the œsophagus and trachea, however, there was external redness. Internally, the œsophagus was evidently somewhat inflamed, as was the mucous membrane lining the trachea; but in neither of these canals was there any distinct trace of effusion.

"Just within the cardiac orifice of the stomach, an effusion of coagulable matter, mediate in appearance between lymph and ropy mucus, had taken place; and that so decidedly as to give the idea of an additional membrane lining the cavity, and visibly terminating at the cardia. This appearance was lost towards the pylorus, by the effused matter insensibly assuming the mucous character. In a few points, the villous membrane presented small spots of capillary vascularity." (P. 190—2.)

We now return to those abdominal affections which, for the sake of perspicuity, we previously passed over. Among these, the most interesting are some observations from the writings of M. Petit, with regard to opening tumors in the region of the liver, supposed to be abscess, but which turned out to be the gall-bladder much distended from obstruction about the neck, and consequent accumulation of its contents. In one case two pints of bile were evacuated, and in another one pint: the operation in both instances proved fatal. The existence of such examples ought to suggest caution to surgeons in opening fluctuating tumors in this region. In another instance, M. Petit being called upon to perform the same operation, divided the skin; but, feeling that the tumor was beginning to subside, the idea struck him that it also might contain bile, which was even then making its escape into the bowels. Under this impression he immediately desisted, and brought the edges of the wound together: no sooner was this accomplished, than a desire to go to stool seized the patient. He passed a large quantity of bile; and in a few days the supposed abscess had disappeared.

A considerable number of curious cases of *biliary calculi* are collected from the works of various pathologists, and allusion made to the more remarkable specimens preserved in different museums: the most striking of these is that of a concretion voided per anum, in the collection of Mr. Heaviside, and which weighs 1320 grains. The manner which calculi, too large to pass by the duct, find their way by ulceration into the duodenum, is described, and followed by some remarks of interest.

"The above facts illustrate so many instances, in which it is most probable that one and the same process, that of ulceration, was called in for the relief of sufferings from the irritation of a large biliary calculus. It has been seen that, even where the gall-stone escapes into the bowel, it is not always able to make its way through the intestines; and that, even where it has made good its passage through that canal, the ulcerated part is so situated that, from its immediate proximity to the stomach, the functions of that organ are disturbed, and so much irritation kept up in the system, that the patient not unfrequently sinks.

"Under these circumstances, nature, ever watchful to remove or to repair the effects of disease, is first observed actively engaged in providing an outlet for the expulsion of the calculus, and then as busily employed in preventing the ill consequences of what has been done, by inducing a gradual contraction of the opening; which in one instance was found so nearly closed, as only to admit a crow-quill. In confirmation of these remarks, I may add that, in a communication with which I was lately favoured by Mr. Brayne, that gentleman says, 'Since the publication of my cases, I have seen two other gall-stones, each larger than either of mine. They occurred in the practice of others, and both patients are alive and well. It is curious to observe how little acute suffering there is in such cases, compared with those in which small calculi pass by the duct.'

"The inconveniences sustained in the digestive functions, by the unrestrained flow of bile from the gall-bladder immediately through the pylorus into the stomach, must be considerable, exciting nausea, sickness, loss of appetite, and other bilious complaints. Neither is it to be expected that the powers of the constitution should be always able perfectly to close the preternatural opening, once made. We see large ulcerated holes from abscesses become smaller, and sometimes remain fistulous: that is, they take on the characters of a regularly organised canal, possessing an inner membrane and surrounding cellular structure. There appears no reason why the same thing should not follow in an ulcerated opening between the gall-bladder and intestinal canal. Again, should the adherent space be small, and the relative position of the parts subject, either of them, to be occasionally drawn away,—as I have frequently, under these circumstances, seen solid adhesions extend themselves,—there seems no difficulty in concluding that adhesions, which are hollow or internally ulcerated, may be subjected to a similar influence; an influence which, in the course of time, must convert a comparatively broad and superficial adhesion, with a central passage, into a narrow elongated tube, the organisation of which will become more perfect the longer it has existed.

"In this way a very singular appearance, noticed in the manuscript of the late Mr. Watson, may be explained. He states that, 'Dec. 26, 1789, Mr. Morell opened the body of a woman, long subject to bilious complaints. The liver was in a diseased and putrid state. The gall-bladder contained many gall-stones: one, lodged in the opening of the cystic duct, had ulcerated the inner membrane. A singular circumstance was noticed about the middle of its internal surface, where a small round hole, or orifice, admitted a common probe directly from the gall-bladder into the stomach, near the pylorus. From hence, probably, the bile had flowed into the stomach, producing some of her complaints. This preternatural passage was a distinct duct, running a full inch, and connecting the two cavities. The neck of the gall-bladder was strongly united to the stomach by inflammatory adhesion: where the duct passed there was no appearance of that kind, the two cavities being an inch distant from each other; that is to say, the whole length of the duct. The preparation was promised to Dr. Baillie.

"The appearances just described are noticed in Dr. Baillie's work, and probably from the same preparation. He says, 'I have once seen an immediate communication, by a short canal, between the gall-bladder and small end of the stomach. This *lusus naturæ* is very rare, and but few instances of it have been recorded." (P. 87—89.)

A few cases of affections of the Pleura are mentioned, but they are not such as to warrant their being quoted.

---

Art. II.—*Medico-Chirurgical Transactions.* Vol. XIII. Part I.—8vo. pp. 280; with five Plates. Longman and Co. London, 1825.

[Continued from page 326.]

11. *Remarks on the Diagnosis, and on the Inversion of the Foot, in Fracture of the Neck and upper Part of the Thigh-bone.* By Mr. Guthrie.

The object of this paper is to settle a point of practice often obscure, and respecting which the best authorities have not given us that precise information upon which a secure diagnosis can be formed. Mr. Guthrie observes, that the nature of the articulation of the thigh, and the manner in which it is surrounded by soft parts, render an examination sometimes difficult; and, therefore, certain accompanying and distinct signs must be attended to, in order to arrive at a knowledge of the particular kind of accident that has taken place. The principal difficulty having been supposed to exist in distinguishing between dislocation of the head and fracture of the neck of the femur: the diagnostic signs usually referred to being common to both, require, therefore, to be considered *collectively*, rather than separately, in order to enable the surgeon to come to a just conclusion.

The dislocations likely to be mistaken for fracture are either that upwards and backwards on the dorsum of the ilium, or backwards only into the ischiatic notch. In the first kind, the great trochanter must be forwards, and nearer to the anterior spinous process of the ilium; the limb is necessarily shortened, from an inch to two and a half inches; the knee is turned inwards, and a little advanced; and the great toe rests on the instep of the other foot. The limb cannot be drawn downwards or outwards, but may be turned inwards; when the head of the bone may usually be felt on the dorsum ilii. The trochanter is less prominent, and the hip, therefore, flatter. In short, the distortion is greater than in any other.

In the dislocation into the ischiatic notch, the limb is not so much shortened, (from half an inch to an inch;) the trochanter major is behind its usual situation; the head of the bone is so buried that it cannot be felt, excepting in thin persons, and then only by rolling the thigh-bone forwards. The knee and foot are turned inwards, but not in so great a degree; and the toe rests against the ball of the great toe of the other foot. The knee is not so much advanced as in the former case; the limb is so fixed that both flexion and rotation are almost prevented.

Mr. Guthrie next mentions another variety of dislocation backwards; but, as he observes that this has not been demonstrated, we shall not enter into its supposed distinctive marks: but we must recollect that dislocation of any kind in the hip-joint is a rare occurrence in old persons.

The above circumstances, then, should always be present to the mind of the surgeon, because fractures of the neck of the femur may be confounded with both the above dislocations, as the limb is made shorter by them. The eversion of the foot and the mobility of the limb in fracture, are said to distinguish the fracture; but the eversion is by no means a constant sign of this injury, and it may be (though that is rarely the case) inverted. Many hours, however, sometimes elapse before the eversion is decidedly apparent. With respect to the fact of the inversion of the foot in fracture, which was first noticed by PARE, it has been disputed by several writers; and lastly by BOYER, who not only never saw it, but finds it difficult to conceive how it ever could take place.

"I had always conceived, with others, and taught in my Lectures, that when the foot was turned in, or neither in nor out, the fracture must have occurred in the cervix of the bone near the head, so that the portion attached to the trochanter passed behind that remaining with the head of the bone, and in this manner caused the turning inwards of the great toe; or that the capsular ligament being torn, it passed backwards behind the acetabulum, giving rise to the same appearance. In either of these hypothetical cases, the inversion of the toe, or point of the foot, should not have continued after the limb had been restored to its proper length; a result which, in the cases I have actually seen, did not follow; proving, therefore, that the opinions entertained of the nature of these cases were unfounded. The only dissection of an injury I am acquainted with, made previously to the one presently to be noticed, in which the toe turned inwards, is in the possession of Mr. Langstaff; but in that case the great toe was, in the first instance, everted, and subsequently turned inwards when the patient began to use the limb. The preparation shows the fracture to have been within the capsular ligament, close to the head of the bone, and gives a decided refutation to the opinion of the length of the broken portion attached to the trochanter being the cause of the inversion, inasmuch as this part has been removed by absorption. The point of the foot was everted whilst it retained its proper length, and only became inverted by a wise provision of nature to assist progression, after it had begun to be shortened. This circumstance receives great illustration in the person of Henry West, a boy from whom Mr. White, of the Westminster Hospital, removed the head, neck, and part of the trochanter, of the left thigh-bone, in consequence of scrofulous disease of the hip-joint attended by abscess. He recovered after the removal of the bone. The thigh is three inches and a half shorter than the other, and the toes turn inwards, not only in walking, but when he lies on his back in a quiescent posture, or prepared for sleep." (P. 108, 109.)

The recital of two cases next follow: in the first of these a lady had slipped, and fallen on her left hip. When placed in a horizontal posture, the limb was a little shorter, and moveable as far as pain would permit the trial; the hip was swollen, and the trochanter was deranged, though in what way could not be distinctly ascertained. There was no crepitus. The injury was declared to be fracture external to the ligament; but, after the first day of treatment, the great toe turned inwards, and continued to do so for several weeks. After an interval of eighteen months, however, the lady could walk with the help of two sticks; and the foot is in its natural position, the limb being a little shorter.

The second case is that of a woman of ninety, who fell from a stool upon her left hip. The limb, when examined, was found rather more than half an inch shorter than the other, and the great toe turned inwards, though not quite so decidedly as in a case of dislocation. The limb was moveable in all directions, but with pain, and it could easily be extended to the same length as the other. No crepitation was observed. The patient died forty-four days after the accident, and a fracture was found external to the capsular ligament: the little trochanter was broken off, and with it the attachment of the psoas and iliacus internus muscles. The head and neck of the femur were diagonally separated from the body of the bone, so as to leave the insertions of the pyriformis, gimellus, obturator externus and internus, and quadratus, with the head and neck of the bone. It will be easily conceived, therefore, that when a fracture takes place *within* the insertion of these muscles, the foot must be everted; but, when it takes place in such a manner as to be *external* to the insertion of these rotators outwards, as in the above case, the toe will be turned inwards. The effect of the breaking off the little trochanter, with the attachment of the psoas and iliacus muscles, must rather have increased this inversion than otherwise.

Mr. Guthrie next makes some observations on the effect of fractures of the thigh-bone below the trochanter, which we pass over, in order to arrive at the inferences which he has deduced as to those accidents, to which he has drawn the attention of the reader more particularly; they are these:—

"1st. That, whilst the eversion of the foot is characteristic of fracture, its absence does not indicate the non-existence of fracture.

"2d. That the inversion of the foot is equally characteristic of fracture as of dislocation, and is only distinguishable, with reference to these two different states, by comparison, or a due estimate of the degree of inversion.

"In the dislocation upwards and forwards, or on the dorsum ilii, the inversion of the foot is complete; the great toe is turned inwards, and rests on the instep of the opposite foot, being the first or greatest degree of inversion. The limb is generally two inches shorter than the other.

"In the dislocation backwards into the ischiatic notch, the inversion of the foot is decidedly marked, but is not so complete as in the preceding case. The knee and great toe turn in, the latter resting against the ball of the great toe of the opposite foot, and not admitting of rotation outwards. The limb is but little shortened, yet cannot be lengthened without great force.

"In fracture, the inversion of the foot is less complete; the great toe merely turns to the opposite one, and sometimes scarcely does this; the limb is but little shortened, is easily everted, readily moved in almost every direction, although not without pain, and is restored to its proper length on the application of a very moderate extension. This constitutes the third degree of inversion.

"3d. That inversion of the foot does not take place in fracture within the capsular ligament; and that this symptom is rather diagnostic of a fracture through the trochanter major, a portion of it being continuous with the shaft of the bone." (P. 114, 115.)

Mr. Guthrie remarks, that the shortening of the leg in a recent fracture of the neck and upper part of the femur will rarely exceed an inch and a half, and, if the limb is measured carefully, will not often be so much. With respect to the greater or less shortening of the limb, with reference to the diagnosis of fracture within or without the capsular ligament, Mr. Guthrie says, that the difference of opinion among surgeons on this point arises from the complicated nature of the injury; since it does not so much depend upon the fracture being within or without the ligament, as upon the precise nature of the fracture: for, if the solution of continuity be not complete, the limb may not be shortened; or the same thing may happen if the bone be broken horizontally, or even diagonally, so that the upper portion prevents the lower one from rising, at least for some days. A great degree of violence, on the contrary, may effect this at once. If the fracture be external to the ligament, but diagonally with reference to the trochanters, the parts may be retained in contact, unless great violence has been applied: hence the greater or less degree of shortening cannot separately form a criterion by which to guide the judgment. As a general rule, however, admitting many exceptions, it may be said that the shortening of the limb, within the first forty-eight hours, is less when the fracture is external to the capsular ligament, than when it is within it. A degree of pain, swelling, soreness, and contusion, would seem to indicate generally a fracture external to, rather than within, the ligament. A crepitus cannot always be distinguished when the limb has been drawn down to its proper length. The change of position in the trochanter is often so trifling as not to be readily ascertained; and the alteration of motion, or rotating the knee inwards, is often unsatisfactory.

The mobility of the limb in fracture, and the contrary in dislocation, have been considered until lately as distinctive marks of these injuries; but Mr. Todd has related a case wherein extensive motion was admitted in a case of dislocation; showing that even on this point the judgment must be formed by comparison. In a case of fracture, motion *can* be given in every direction, though attended with great pain. In dislocation, motion cannot be given to the limb in the same extent; and least of all towards complete abduction. Finally, in dislocation, the shortened limb cannot be restored to its proper length without the employment of great force; or rather, in fracture, this can be accomplished by the surgeon alone; and, when so restored, it is soon retracted or shortened as much as before.

We have shortened Mr. Guthrie's paper; but, on a practical point of so much importance, have thought it better to give as fully as we were able the whole of his arguments, without mingling them with any remarks of our own.

12. ***On the Effects of Loss of Blood.*** By Dr. Marshall Hall.

The subject of this paper is of the highest interest, both in a physiological and practical point of view, and we recommend it as meriting particular attention: it is the most important in the volume. It is stated by Dr. Hall, and justly, that the effects of loss of blood are frequently such as suggest the idea of increased power of the system; thence leading us to the further employment of the lancet, under circumstances when its use is highly prejudicial. As an example, we may refer to the throbbing of the carotids, and apparent determination to the head, which follow uterine hemorrhage, or any other very copious depletion; a state of symptoms leading an inexperienced practitioner to the idea that still further evacuation is required to free the system from the increased arterial action. This we have had occasion to see, and therefore conclude that others are equally familiar with the fact. But we must return to the author before us. In giving us the results of his observations on the effects of loss of blood, he adopts the following arrangement:—

"I. Of the immediate effects of loss of blood, chiefly syncope, and of the re-action or failure of the vital powers.

"II. Of the more remote or cumulative effects of repeated or protracted loss of blood, or *exhaustion:* and, 1, of exhaustion with excessive re-action; 2, of exhaustion with defective re-action; 2, of exhaustion with sinking.

"III. Of the effects of further loss of blood in cases of exhaustion. 1. Of the substitution of syncope for re-action; 2, of the transition of the state of re-action into that of sinking; 3, of sudden dissolution.

"IV. Of the influence of various circumstances on the effects of loss of blood. 1. Of age, &c.; 2, of disease.

"V. Of the effects of loss of blood on the internal organs. 1. The brain; 2, the heart; 3, the lungs; 4, the intestinal canal, &c."

I. With regard to the immediate effect of loss of blood, the most common is syncope. In this condition the phenomena seem to indicate that the brain is the part the function of which is first impaired; next the respiration suffers, and the action of the heart becomes weakened, both from the diminished quantity of blood brought to it, and from the difficulty in its arterialisation.

"In cases of profuse hemorrhagy, the state of the patient varies: there is at one moment a degree of syncope, then a partial recovery. During the syncope, the countenance is extremely pallid; there is more or less insensibility; the respiratory movements of the thorax are at one period imperceptible, and then there are irregular sighs; the pulse is slow, feeble, or not to be distinguished; the extremities are cold, and the stomach is frequently affected with sickness. I have observed that when the movements of the chest, in the interval between the sighs, have been imperceptible or nearly so, the respiration has still been carried on by means of the diaphragm. It may also be remarked, that the state of syncope is often relieved, for a time, by an attack of sickness and vomiting; immediately after which, the patient expresses himself as feeling better; the countenance is somewhat improved, the breathing more natural, and the pulse stronger and more frequent.

"In cases of fatal hemorrhagy, there are none of these ameliorations. The symptoms gradually and progressively assume a more and more frightful aspect. The countenance does not improve, but becomes pale and sunk. The consciousness sometimes remains until towards the last, when there is some delirium; but every thing denotes an impaired state of the energies of the brain. The breathing becomes stertorous, and at length gasping. The pulse is extremely feeble, or even imperceptible. Animal heat fails, and the extremities become colder and colder, in spite of every kind of external warmth. The voice may be strong, but there is constant restlessness and jactitation. Ultimately the strength fails, and the patient sinks, gasps, and expires." (P. 124—5.)

The system usually rallies spontaneously from syncope; and the act by which the recovery is effected, is called re-action. Now this re-action may be either excessive or defective, or altogether wanting. The phenomena resulting from these circumstances form the subject of subsequent inquiry: at present it is only to be added, that loss of blood occasionally gives rise to attacks of convulsions, instead of syncope.

II. The recovery, under favourable circumstances, is generally a simple return to health, the pulse not exceeding its natural frequency. When the bleeding has been profuse, the pulse becomes, and continues for some time, more frequent than natural; and if the individual, instead of a copious loss of blood occurring at one time, is subjected to repeated venesection, the pulse acquires an increased frequency, with throbbing, and occasionally all the symptoms of excessive re-action. They are thus described:

"The state of excessive re-action is formed gradually, and consists, at first, in forcible beating of the pulse, of the carotids, and of the heart, accompanied by a sense of throbbing in the head, of palpitation of the heart, and eventually, perhaps, of beating or throbbing in the scrobiculus cordis, and in the course of the aorta. This state of re-action is augmented occasionally by a turbulent dream, mental agitation, or bodily exertion. At other times it is modified by a temporary faintness or syncope. In the more exquisite cases of excessive re-action, the symptoms are still more strongly marked. The beating of the temples is now accompanied by a throbbing pain of the head, and the energies and sensibilities of the brain are morbidly augmented. Sometimes there is intolerance of light, but still more frequently intolerance of noise and disturbances of any kind, requiring stillness to be strictly enjoined, the knockers to be tied, and straw to be strewed along the pavement. The sleep is agitated and disturbed by fearful dreams; and the patient is liable to awake in a state of great hurry of mind, sometimes almost approaching to delirium. In general this is slight, but occasionally severe and even continued. More frequently there are great noises in the head, as of singing, of crackers, of a storm, or of a cataract; in some instances, flashes of light are seen. Sometimes there is a sense of great pressure, or tightness, in one part or round the head, as if the skull were pressed by an iron nail, or bound by an iron hoop.

"The action of the heart and arteries is morbidly increased; and there occur great palpitation and visible throbbing of the carotids, and sometimes even of the abdominal aorta, augmented to a still greater degree by every hurry of mind or exertion of the body, by sudden noises or hurried dreams and wakings. The patient is often greatly alarmed, and impressed with the feeling of approaching dissolution. The state of palpitation and throbbing are apt to change, at different times, into a feeling of syncope. The effect of sleep is in some instances very extraordinary; sometimes palpitation, at other times a degree of syncope, or an overwhelming feeling of dissolution. The pulse varies from 100 to 120 or 130, and is attended with a forcible jerk or bounding of the artery.

"The respiration is apt to be frequent and hurried, and attended with alternate panting and sighing. The movement of expiration is sometimes obviously and singularly blended with a movement communicated by the beat of the heart; the patient requires the smelling bottle, the fan, and fresh air. In this state of exhaustion, sudden dissolution has sometimes been the immediate consequence of muscular effort on the part of the patient.

"The phenomena of excessive re-action are mostly observed in young persons of robust constitution, who have been subjected to repeated blood-letting. In infants, in feeble persons, and in rather advanced years, re-action after loss of blood is for the most part defective. In this case the patient long remains pale, thin, and feeble, and becomes faint on the slightest occasions: the pulse is frequent, but feeble, and perhaps irregular; and we look in vain for the throbbing and palpitation observed in the young and robust. This state either gradually yields to returning strength, or subsides into the state of sinking. In the study of the effects of loss of blood, it is particularly necessary to bear in mind this difference of the phenomena arising out of the previous state of the constitution, whether of vigour or of feebleness." (P. 127—129.)

These symptoms, indicative of exhaustion with excessive re-action, may gradually pass away, or they may give place to *sinking*. In this, the sensibility is diminished, there is a tendency to dozing, with snoring; stertor, or blowing-up of the cheeks in breathing, a symptom which all must have observed in apoplectic affections. The patient is inattentive to what is passing around him; forgets his situation; requires a moment to recollect himself when spoken to, and soon relapses into dozing. In addition to these circumstances, our author has remarked "a crepitus in respiration, only to be heard at first on the most attentive listening:" this soon increases, passing into rattling in the throat. There is some degree of labour in the breathing, inducing acuteness in the nostrils, and occasionally a peculiar cough. The heart loses its palpitation, and the arteries their throbbing; the stomach and bowels become affected with flatulence; the will loses its command over the sphincters; delirium, jactitation, and coldness of the extremities, mark the approach of dissolution. A very interesting and important case, illustrates some of the phenomena above mentioned, which, however, is unfortunately too long for quotation.

III. Dr. Hall next considers the effect of further loss of blood in cases of exhaustion, and states his belief that the symptoms of re-action have frequently been mistaken for those of inflammation of the head or heart; under which impression, patients have been bled still further, the effect of this being the temporary relief of the symptoms. Here, as throughout the paper, we mark and bear testimony to the accuracy of the author's observations. It was some time before he could comprehend the nature of this fact, but at length he perceived that the symptoms relieved were those of re-action, and that the mode of relief consisted in the substitution of syncope. Yet, although relief be given for a time, the remedy eventually adds to the severity of the malady, in the majority of cases. It is to be observed, that re-action seldom supervenes from one depletion, however copious; while the repetition of this is very apt to produce it. When re-action has supervened, syncope very readily follows any further loss of blood. "This fact is of importance, because it may be regarded as a sign of the state of exhaustion when obscured by the re-action of the system, and as a warning voice against further and inconsiderate use of the lancet." When the depletion is still further repeated, then not merely syncope, but sinking, comes on, and with it the train of phenomena already described.

IV. This section relates to the influence of various circumstances on the effects resulting from loss of blood. Of these, the strength is the first and most important, the degree of re-action being in proportion to it. In feeble constitutions, as in infancy and declining years, the re-action is defective, and the state of syncope is one of danger, so that a repetition of the bleeding is borne with difficulty. Just the reverse of this occurs in the robust when the re-action is strong: in these, indeed, the state of sinking is preceded by great re-action, unless the strength be overwhelmed by the extent of the evacuation. Other circumstances exerting an influence over the effects of blood-letting, are certain diseases; and it is particularly stated by the author, that "intestinal irritation leads to those effects of blood-letting which I have described as exhaustion; whilst inflammation seems to protect the system from the effects of loss of blood:" so that, taking health as the medium, the presence of intestinal irritation would lead to the more speedy, and of inflammation to the more tardy, occurrence of exhaustion.

V. The effect of loss of blood on the internal organs is very little known. The author conjectures that effusion into the ventricles of the brain takes place; and that there is, "even in cases of exhaustion from loss of blood, increased action or fulness of the vessels of the brain." There is certainly, in cases of great exhaustion from any evacuation, a tendency to serous effusion throughout the cellular membrane generally.

Dr. Hall purposes to investigate the *organic effects*, and especially the *remedies*, of loss of blood. To these we look forward with the anticipation of additional information upon this interesting subject.

The remaining contents of the volume consist of little more than cases, such as usually are, and ought to be, published in Medical Journals, rather than in the Transactions of a learned Society. Some of these we shall probably give in our COLLECTANEA on some future occasion.

---

### DIVISION II.

### FOREIGN.

ART. III.—*Recherches Statistiques sur la Durée moyenne des Fièvres Intermittentes. Memoire lu a l'Institut.* Par M. BAILLY, de Blois, D.M.P.

*Statistical Researches on the mean Duration of Intermittent Fever.* By M. BAILLY, of Blois, D.M.P.

Two principal sources of arriving at the real knowledge of diseases exist. The first is clinical observations of the symptoms which manifest themselves in the progress of a malady, compared with the organic alterations which are met with in the bodies of those who fall victims to it: the second, not less important than the first, consists in the study of these diseases in great numbers, and the general results which this study makes known to us. These last, exempt from all the exceptions and peculiarities which isolated cases afford, are much more proper to discover the organic laws which regulate their existence. The causes of every vital action which is executed in the human machine not only vary in activity in different individuals, but also in the same individuals placed under different circumstances; and this variety sometimes proceeds to the extent of concealing even the general properties common to all mankind. Observations minutely made at the bedside of the patient sometimes give to mere accidents and exceptions, which exist in these single cases, an importance equal, or even sometimes superior, to that of the fundamental laws of our organisation; but, in extending our researches to a great number of individuals, exceptions are destroyed: the most constant phenomena become remarkable by their permanence, and precise notions are acquired respecting general phenomena, the intimate nature of which we wish to be acquainted with.

Intermittent fever has never been studied in France by means of these two proceedings: the rarity of the disease sufficiently accounts for this. Italy has always afforded a more favourable field for this study; but, at the same time, there have always existed obstacles which have not been easy to surmount. The situation of an hospital in precisely the most unhealthy part of Rome, and the danger of making post-mortem examinations in the heat of summer, have been the chief of these; and, in consequence, we find, among the physicians of Rome and the other marshy situations of Italy, numerous reports of cases of cure, but few accounts of the examination of the dead: and thus it is that neither LANCISI nor BAGLIVI, whose opportunities of observation were so great, have made any progress in the inquiry. TORTI, whose researches with respect to malignant intermittent fevers are perhaps amongst the most precious which we possess, has not dared to give them the perfection which his residence at Rome might have permitted him to do. I must say the same of RAMAZZINI, of Modena, and all the other Italian physicians, who have feared to encounter the dangers which autoptic examinations are fraught with, where the climate, as well as local circumstances, are so peculiarly adverse to them.

In the first memoir which M. BAILLY read before the Academy of Medicine, he gave a sketch of the cases collected in the hospitals at Rome, together with the examinations after death. The present memoir contains the result of his observations on these diseases, made among a number of individuals sufficiently large to neutralise, as it were, the exceptions, and to make apparent the general law of the organisation subject to these affections.

The hospital of St. Esprit at Rome contains, during the greater part of the year, scarcely any patients excepting those affected with intermittent fever. The cinchona is administered daily to many hundreds of sick, and generally each of them takes this medicine from the day of his admission to that of his discharge. Now it appears, by the tables furnished from the registers of this hospital, that the mean period of sojourn in the hospital of each individual is about a fortnight. This is at least the product of an examination of the cases of about 60,000 patients. Admitting, then, that the type of intermittent was tertian, which is by far the most common, each individual may have had seven or eight paroxysms.

In a Report published at Lyons on the intermittents treated in the hospital of that city, from 1806 to 1812, mention is made of nearly 600 cases; more than three-quarters of whom were cured without bark: apozems and purgatives constituted almost entirely the therapeutical means employed in these cases. Now, the mean duration of the complaint in each individual, local circumstances excepted, has equally been about two weeks.

In 1778, Dr. CAUSLAND, having occasion to treat many soldiers affected with ague in Canada, and having expended his stock of cinchona, conceived the idea of substituting the tartar emetic, at first by itself, afterwards united with opium. In reviewing the period required for the cure, he found that their duration, (the type in America being generally either quotidian or double tertian,) was about eight paroxysms; that is, nearly a fortnight. Of late years, Dr. PEYSSON has proposed the substitution of the same means (tartar emetic united with opium,) instead of the bark; and this form of preparation was also formerly employed at Montpellier. A great number of patients were treated with this potion; but, counting the number of paroxysms which preceded the administration of this remedy, and adding the number of those which succeeded, the same result is obtained: that is, the patients have each had about eight attacks, which have occupied either one or two weeks, depending upon whether the type has been simply tertian or double tertian.

Thus, these fevers, treated as at Rome with the cinchona; those of Lyons, treated without it; the cases in America, in which the emetic tartar and opium was administered; and those under the care of Dr. Peysson, have all presented a duration of a fortnight, and have all required the same residence in the hospital of one or two weeks, according to the type of intermittent which prevailed.

Intermittents, then, considered in examples of thousands of individuals, are not nervous affections, susceptible of being arrested by medicine at our pleasure: they have a regular course to follow, and periods to run; and, although they terminate under the influence of certain remedies, to which all the honour of the cure is attributed, they only, in fact, yield spontaneously to those medicines which succeed, merely because the disease was about to cease of its own accord.

It will easily be conceived that this fact is of great practical importance. We now proceed to examine upon what physiological principles it is founded. Hippocrates, and all observers since his time, have remarked that acute diseases had a mean duration of a fortnight: there is, therefore, something common between acute diseases and intermittents; and this is exactly what the numerous researches which M. Bailly has made in morbid anatomy has demonstrated, since having opened nearly the whole of those who died in the hospital of St. Esprit at Rome in the summer of the year 1822, he constantly found marked indications of inflammation of some of the principal organs. M. Bailly is, however, far from thinking or asserting that therefore ague is nothing but inflammation: but, having shown the incontestable fact that, whatever the treatment adopted may be, that disease has a duration invariably constant, and that this duration is precisely that met with in acute diseases, and moreover that those lesions constituting acute disease are essential to it; it appears to him that, without going beyond the truth, it may be affirmed that, in intermittent fever, the return and number of the paroxysms is, in the greater number of cases, under the influence of an internal inflammation, which is opposed to the cessation of the febrile action, until it has fully completed its course. When the inflammation is dissipated, then the bark succeeds in opposing the return of the nervous phenomena which constitute the febrile paroxysm. But if this movement of the nerves, whatever be the epoch in which we endeavour to suppress it, must necessarily return for a definite number of times, according to those organic laws which regulate the termination of acute or inflammatory diseases, it is entirely useless to administer either the cinchona or any other febrifuge, as is the custom with many practitioners from the very outset of the complaint: for the tables of mortality drawn from the records of the hospitals of Rome prove that the utmost we can expect by this method is to find that the fever will resist all the remedies opposed to it, and to pursue the number of revolutions assigned to it, in spite of them.

It is not unusual to see the sudden suppression of the paroxysms give rise to serious accidents, or produce chronic diseases; which, although not immediately mortal, never permit the patient to resume his usual state of health. Ramazzini, resting his arguments upon cases of this nature, gave an appearance of reasonableness to the vivid declamations which he has published against the use of the bark; the consequences arising from which would probably have been adopted by most practitioners, if he had not applied his interdiction of this remedy to those affections which, like the malignant kinds of intermittent, are altogether different from the more simple species of ague, both in relation to their duration and the treatment they require. Besides this, the dangers resulting from a sudden suppression of ague have been too long recognised to render it necessary to insist upon them; but it was of importance to determine by statistic observations, and by the examination of bodies, the reality of the facts and the soundness of the deductions drawn from them; especially at a period like the present, when we begin to doubt even the facts seen by our predecessors, since they have not been observed in connexion with the present existing principles of physiology. It becomes, then, evident that the suppression of a paroxysm not diminishing in any degree the state of internal inflammation, only prevents it from exhibiting itself in general symptoms; since the force which determines the fever is merely paralysed; and then this inflammation, not being able to continue its course without exercising a hurtful influence upon the economy, gives rise to sympathetic and secondary affections, which, although they advance imperceptibly, do not terminate less certainly in giving birth to lesions that are irremediable.

If physicians have generally admitted that certain diseases always preserved a mean duration nearly equal, no one has sought to develope the physiological cause of this duration; nevertheless, it might have occurred to us that this knowledge alone could form the sure basis of practical medicine. To have discovered why a disease lasts a specific time, is it not to have travelled at least half the road necessary to make us acquainted with the whole of the phenomena? Nothing more remains than to discover the means to modify that which gives rise to this duration of the disease.

In order to understand the causes of so singular a fact, let us examine the most simple case, and one of those on which all physicians are agreed. What is a pleurisy? There is scarcely any person, perhaps, who does not believe that he possesses a clear and distinct idea of this affection, and who is not content to look upon it as an inflammation of the pleura. This definition brings with it a notion so decidedly fixed in the opinion of all the world, that no one seems disposed to find in it the uncertainty that the greater part of all other kinds of maladies bring with them. This happy disposition of the mind, which exists regarding the nature of the disease, equally belongs to the treatment which it demands. Every one bleeds, more or less abundantly, patients seized with these affections, if they exist in a certain degree of intensity. All medical men know that, on opening the bodies of those who die of this disease, such phenomena as the injection of blood-vessels, bloody extravasations, suppurations of the pleura, &c. are met with, and that they indicate a greater activity of the circulation, and the other organic functions of that membrane. From all these united circumstances results a sentiment that induces us to regard the presence of blood in the tissue of the pleura as the cause of all the evil; and this belief is applied to the knowledge and treatment of all inflammations. To remove the cause, blood is taken away: and if, by this method, as is frequently the case, success is obtained, nothing induces us to reflect anew upon a case which is considered as sufficiently understood, since we have obtained the end desired.

Let us now see to what extent these general ideas are well founded; and let us inquire whether the presence of blood in our tissues is the fundamental cause of an inflammation, and of its duration. It is supposed that a common-sized man has usually about fifteen pounds of blood: he enjoys good health with this quantity, and this therefore constitutes his condition physiologically. A sudden cold produces a violent pleurisy. Behold him, therefore, dangerously ill, with the same quantity of blood which he possessed some moments before, when he was in perfect health. He is bled several times: four, six, or eight pounds of blood are successively taken from him, in so short a time that the animal economy cannot repair its losses. The pleurisy, nevertheless, shall go on constantly augmenting, and a patient falls a victim to an inflammation which indicates an excess of blood, and a vital activity greater than in a state of health, although the mass of liquids has been diminished a quarter, a third, or perhaps even one half. But it may be said that the blood has been amassed in the pleura, at the expence of all the system, which has been deprived of it; and that the diminution caused by the bleedings have not been sufficient to take from the vessels of the pleura all the blood which has been carried there in excess. This objection falls to the ground of itself, at the very sight of a patient suffering under a violent attack of fever, which propels the fluids into all the vessels of the interior, as well as of the surface of the body, with an activity unknown in a state of health. The head, the abdomen, and the skin, are certainly the seat of a sanguineous congestion, much more powerful during the existence of a pleurisy accompanied with fever, than during the healthy state. The system, then, is not deprived of blood during the course of inflammations, which are far from determining a *raptus* of that fluid to a circumscribed spot. To prove that the presence of blood is not the cause of inflammation, it is only necessary to cite those cases known to all practitioners, and in which, when a pleurisy is thoroughly established, all the blood in the body may be taken away at one bleeding without removing the disease: the patient would then die exsanguineous; and, at the opening of the body, it would be found that the pleurisy had not disappeared. It cannot, then, be affirmed that he was cured of the pleurisy when he died; as might have been the case had the disease merely consisted of a simple congestion.

Now, therefore, as it appears that, whatever quantity of blood is taken away from a patient who dies of a pleurisy, this affection does not disappear, if the greatest number of inflammations properly treated are not cured immediately upon the abstraction of the blood,—if they, of necessity, last a given time, as is shown by observations made in every age and at every place,—if they can destroy a patient, from whom, during his disease, the greater part of the fluids of his body have been taken, it must be concluded that these affections are not entirely the result of an excess of blood in the diseased part; and that the organic change which has taken place in an inflamed part is independent of the blood that circulates therein, though the alteration has perhaps been produced through the medium of that fluid. Finally, that this change requires many days to disappear, since a few seconds are sufficient to produce the most considerable sanguineous depletions.

An inflammation once established consists, then, in a fixed permanent alteration, in a modification of the nutrition of the diseased tissue. An organ that is inflamed acquires, therefore, during the whole time that the disease takes to develope itself, properties different from those which its ordinary nutrition affords; and, as these acts of nutrition are slow and successive,—as they consist of a change in the molecules which are brought to and carried from them, in a continued manner,—every inflammation, which is nothing more than the same labour performed with materials somewhat modified, must of necessity occupy a certain time to acquire its maximum of intensity, and to disappear completely.

To make us understand in what manner he views the organic operation in which the essence of inflammation consists, M. Bailly observes, that this pathological phenomenon is a modification of nutrition, analogous, as far as regards the manner in which it is effected, to that which takes place in the bones of those animals fed with madder. The new red molecules, which are interposed among the osseous molecules of animals subjected to these experiments, require several days to give to the bones the highest degree of colour which they are able to receive; and, when ordinary nourishment is substituted for the madder, the red molecules disappear slowly, and require a determinate time to pass away, and to enable the bone to return to its former condition. Now, in the same way that the colour of the bones does not disappear suddenly by a total subtraction of the blood of the animal, in like manner the peculiar modification which an inflamed organ has acquired by nutrition is a fixture *(fixité)* which requires a certain time to destroy. It is necessary in both cases that the successive renovations of the parts in the organs diseased, or modified in their nutrition, resume the new properties which particular influences have impressed upon them. It is nutrition that has altered the inflamed tissue: it is nutrition which is charged with the reparation of the evil it has produced. Without doubt, the influence of the circulation, and the presence of the blood in an inflamed organ, is very great, since it is this fluid which is charged with the deposition of the molecules which renew the composition and

2

constitute the properties of our tissues; but it is also easy to conceive that, when it has deposited, during a certain number of organic revolutions, materials of a particular nature in a diseased part, it may be suddenly subtracted from that part, although the evacuation does not take away suddenly the composition which was acquired by a series of acts of nutrition.

M. Bailly does not here speak of those inflammations which are overcome suddenly, as it were, by an energetic treatment. Simple sanguineous congestions have, under the same name, been certainly confounded with fixed inflammations of the nature of those which have been described. It is true that all congestions may, if they continue a certain time, produce a real inflammation; but, as long as it only consists in a sanguineous fulness, which has not changed the nutrition of an organ, it may suddenly disappear in consequence of abundant bleeding and derivatives, which change the direction of the fluxionary movement. All that has been said, therefore, only applies to those inflammations well established, and which necessarily occupy some days in disappearing. It is equally upon this view of the physiological phenomena that the permanency and necessary duration of acute maladies and inflammations is explained, that the theory of the mode in which bleeding operates is founded; the proper use of which must be uncertain and irregular, when we are ignorant by what organic laws it tends to the cure of these diseases.

If the theory of nutrition indicates to us the necessity of a certain number of organic revolutions to produce and to destroy an inflammation, experience alone would teach us how many of these revolutions were necessary to produce and to get rid of such a phenomenon. It is this direct observation which has taught us that this duration was one week in the majority of slight eruptive diseases; whilst acute complaints run their course in fourteen days, or two weeks. It is this also which has discovered that affections, so irregular in appearance as intermittent fevers, were subject to the same necessary laws as acute diseases; and that, by their duration, they belonged to either one or the other of these classes of affections: that is, that they could arrive at their termination either in seven or fourteen days, according to their degree of intensity.

This singular tendency in the animal economy to proceed thus from one week to another in the execution of morbid phenomena, has certainly something astonishing in it; but is not the healthy state of the body also subjected to certain kinds of periodical movements or organic circuits, which announce a regularity of movement, even where our superficial observation leads us to imagine that these events succeed each other without order, or without any connexion between them? Menstruation takes place every twenty-eight days; that is to say, at the end of four weeks. Is it, then, more easy to conceive that organic phenomena may have their termination in seven or fourteen days? The appearance of the teeth,—the age of puberty,—the return of menstruation in the female,—are they not, like the mean duration of disease, so many primitive facts in the animal economy, proved by observation, and inexplicable as far as regards their first causes?

The physiological theory of the duration of diseases being made out, it will not be difficult to make the application to the study of other affections; and it is not to be doubted but that results will be obtained equally valuable in physiology and in pathology. To prove that two weekly revolutions are necessary to produce the termination of acute diseases, is, in fact, merely to prove that the change of nutrition which constitutes inflammations of the mucous membranes, require fourteen days to produce them, and to cause their disappearance; for it is known that, in general, the ancients only admitted, under the denomination of acute diseases, fevers and other inflammations having their seat in the organic system. But each living system must necessarily present some difference in its mode of nutrition, and consequently in the time required to produce disease. Thus, the serous membranes, for example, the periosteum and other fibrous membranes, the nervous, bony, and muscular tissues, &c. do not demand an equal time with others to produce inflammation; and, although we have some few hints relative to this subject, nevertheless, as it has never been possible to collect documents sufficiently extended to explain this precisely, it is to be hoped that further observation will supply what is still wanting in physiology and pathological anatomy under this head. It is to be wished that the physicians of hospitals would occupy themselves in making out statistic tables, in which the mean period of residence in an hospital of each individual labouring under the same disease should be shown: for those published in France, giving only the mean duration of residence of the whole of the patients of an hospital, are completely useless; this duration not pointing out any one disease in particular, but a mixture of all patients labouring under the most dissimilar complaints. The hospitals of Rome do not labour under this inconvenience; each of them only receiving those diseases, such as intermittent fevers and pleurisies, having a course nearly the same, but more regular than in France; and therefore they give, in the general change that takes place in the hospital, a mean result, which differs but little from the mean duration peculiar to each affection. "It is to be hoped, then," says M. Bailly, "that some one will effect in other diseases what I have performed in intermittent fever, and that, to researches instituted in the cases of single individuals, may be united general tables, obtained by the observation of many thousands of sick persons. The first class indicates how the disease runs its course, when studied in its elementary parts; whilst the general results show us the time employed in effecting these objects,—or, rather, the law of organisation disengaged from any individual influence."

The knowledge of the mean duration of diseases in general, and of intermittent fevers in particular, is not only precious in a physiological point of view, but it offers important advantages to pathology. From what has been said above, the following conclusions may be drawn:—

1. That intermittent fevers are composed of the phenomena of inflammation, together with a general nervous movement, which constitutes the paroxysm properly so called.

2. That these inflammatory phenomena, consisting in a change of nutrition, necessarily present a certain duration founded upon the physiological fact of the composition and decomposition of our organs.

3. That the renewal of the molecules of different parts by nutrition, operating by slow and successive steps, is the physiological cause of the necessary duration of inflammations, and other acute diseases.

4. That the numerous examinations of dead bodies agrees with the tables of admissions and discharges in the hospitals of Rome, and permits us to arrange ague among the number of acute diseases; and, by the statistical tables, it is proved that their mean duration is the same that has always been acknowledged in those affections.

5. That the best method of abridging the duration of intermittents, is to combat the state of internal inflammation.

6. That the nervous movement which constitutes the febrile paroxysm, and which may be attacked by the bark, generally resists the effects of this medicine, until the internal inflammation has run its course.

7. That in simple cases it is sufficient to destroy this inflammation, in order to cure the disease, which, if it persist afterwards by the result of habit, is afterwards easily dissipated by medicines which break this habit, and which are then administered with the greatest success.

8. That the sudden suppression of the febrile action by febrifuge medicines, not destroying the inflammation which exists at the same time: this, instead of re-acting upon the sanguineous and nervous systems, so as to develope the fever, acts silently upon the internal organs, and ends by producing serious lesions; which, although they may not produce very apparent symptoms, do not the less surely undermine the general health, by determining irremediable organic changes.

9. That the administration of antiperiodic medicines at the commencement of the disease, is attended with the inconvenience of making the patient swallow large doses of medicines, the last of which only can be efficacious.

10. That, by giving febrifuges only towards the termination of the disease, not only do we avoid the risk of troubling the regular course of the affection, but we have besides the advantage of terminating it with very small doses.

11. That this method diminishes very much the chances of relapse, so frequent in those cases where we have only endeavoured to attack the febrile paroxysm.

12th and lastly. It diminishes greatly the expence of a treatment always too dear for the indigent classes, when obliged to employ a quantity of the sulphate of quinine.

---

